# Locally Recurrent Leiomyoma of the Bladder Refractory to Visually Complete Transurethral Resection: An Indication for Cystoprostatectomy

**DOI:** 10.1155/2019/1086575

**Published:** 2019-08-25

**Authors:** Katharina Mitchell, John Barnard, Adam Luchey

**Affiliations:** ^1^West Virginia University School of Medicine, Morgantown, WV, USA; ^2^West Virginia University, Department of Urology, Morgantown, WV, USA

## Abstract

Leiomyomas are benign smooth muscle tumors that have low malignant potential (0.1%) and can arise in nearly any area of the body. Genitourinary involvement is very rare and represents only 0.05% of all bladder tumors (Mendes et al., 2017; GÖK, 2017). The most common presenting symptoms of bladder leiomyomas are obstructive voiding (49%), irritative voiding (38%), and hematuria (11%) (Goluboff et al., 1994). Treatment involves complete excision, in this case transurethral resection (TUR), and generally results in complete cure with no recurrences noted in the 250 cases reported in the literature for open resection and 18% recurrence rates after TUR which were successfully treated with a repeat TUR in all cases. Herein, we report a case of leiomyoma of the bladder which was refractory to four visually complete transurethral resections and ultimately required radical cystoprostatectomy with ileal conduit urinary diversion.

## 1. Introduction

Bladder leiomyomas are very rare, representing only 0.05% of all bladder tumors. Worldwide this equates to a 250 cases total ever reported [1, 2]. Equal incidence has been reported between males and females. While leiomyomas of the bladder are benign, they are locally invasive and often symptomatic. The most common presenting symptoms are obstructive voiding (49%), irritative symptoms (38%), and hematuria (11%) [3]. While rare, bladder leiomyomas have historically been cured with surgical extirpation.

## 2. Case

**Figure 1:**
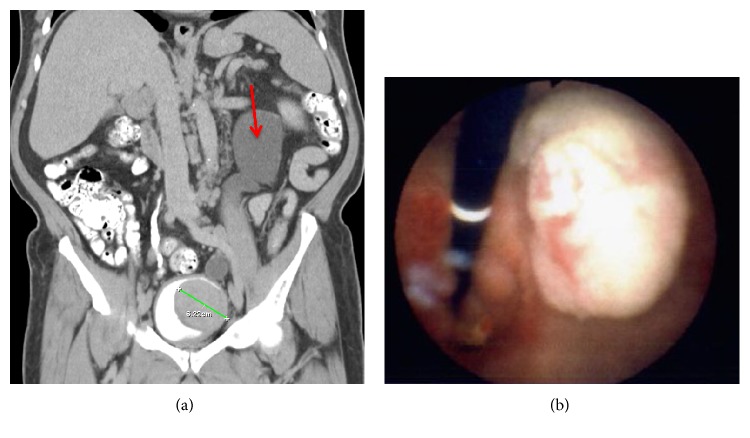
(a) CT Urogram, delayed phase, coronal view, showing 6 cm intravesical tumor and left sided hydroureteronephrosis (red arrow) due to mass effect at the level of the left ureteral orifice. (b) Cystourethroscopy revealed a pale white to tan exophytic bladder mass overlying the left ureteral orifice which was obscuring and obstructing the left kidney and ureter.

A 64-year-old male with a past medical history of coronary artery disease, cardiac stent, COPD, HTN, and dyslipidemia presented as a referral for left testicular teratoma and primary bladder leiomyoma. Prior to presenting to our facility, he underwent left radical orchiectomy, and hernia repair due to enlarging left testicle, had a transurethral resection of bladder tumor (TURBT) due to hematuria, and underwent PSA screening. Pathology determined the testicular mass to be a pT1 teratoma without lymphovascular invasion, PSA was found to be elevated (was 4.2 in 2014, now 4.39), and the 40 g of bladder tumor resected during the TURBT was found to be a leiomyoma of the bladder showing no sarcomatoid differentiation. A CT scan was performed which was notable for bilateral pelvic adenopathy with lymph nodes greater than 2 cm and an interaortocaval lymph node of approximately 1.2 cm. Additionally, outside CT scan with IV contrast of abdomen pelvis showed large 6 cm heterogeneous soft tissue mass within the posterior left aspect of the bladder (Figure 1(a)). The mass was causing complete obstruction of the left ureterovesical junction (UVJ), which resulted in severe left sided hydronephrosis and hydroureter. Although the CT findings were highly concerning for metastatic process, the TURBT specimen did not support the diagnosis of metastatic disease from a bladder primary as it had returned leiomyoma of bladder without sarcomatoid elements. The disparity between imaging findings and pathology results prompted confirmatory testing on both the initial testicular mass and the TURBT specimens. After discussion at multidisciplinary GU tumor board, the consensus recommendation was for CT-guided lymph node biopsy, repeat transurethral resection of bladder tumor, and decompression of the left upper tract via ureteral stent, or nephrostomy. The patient underwent rigid cystourethroscopy, monopolar transurethral resection of bladder tumor (large >5 cm) and monopolar transurethral resection of prostate. During this surgery, a large tumor located on the left wall extending down into the bladder neck and prostatic urethra was resected with an extensive amount of tissue removed (Figure 1(b)). The right ureteral orifice was uninvolved, but the left ureteral orifice was completely obscured by tumor. The bladder neck and prostate were resected down to the level of the verumontanum which was spared. A 3-way foley catheter was placed at the conclusion of the case and the patient was admitted post operatively. He underwent left percutaneous nephrostomy (PCN) placement and IR guided biopsy of the suspicious pelvic lymph nodes prior to discharge.

Following independent pathology review of the outside facility testicular specimen and review of in-house specimens, the previous diagnosis of testicular teratoma was recharacterized as a Leydig cell tumor of the testis and the patient's lymph node biopsy revealed follicular lymphoma. Discussion at multidisciplinary tumor board initially recommended bone marrow transplant, neoadjuvant chemotherapy, and extirpative surgery at a future date depending on the patient's response to chemotherapy. After bone marrow biopsy, his follicular lymphoma was staged as stage 4 with minimal bone marrow involvement. In advanced stage follicular lymphoma there is no survival benefit to early initiation of treatment, therefore serial PET CT scans 6 months apart were recommended by hematology/oncology to follow his lymphoma.

The patient was managed conservatively with nephrostomy tube exchanges every 10–12 weeks and underwent cystoscopy with urodynamic studies after presenting with urinary retention and traumatic foley at approximately 1 year post resection. Cystoscopy revealed a large mass in the left lateral bladder wall extending down into the bladder neck and involving the prostate. He was started on Flomax and discharged without a foley catheter as he was able to void with a normal PVR at the time of the procedure. Due to significant regrowth of leimyoma on cystoscopy, he was encouraged to have repeat debulking of the tumor via transurethral approach as he declined cystoprostatectomy. The patient underwent repeat TURBT with bipolar resectoscope for recurrent left lateral bladder wall, bladder neck, and prostatic mass. The pathology again returned leiomyoma without sarcomatoid differentiation. A month after discharge the patient presented to an outside Emergency Room after developing gross hematuria. He was transferred for continuous bladder irrigation and refractory hematuria which required urgent TURBT to fulgurate bleeding vessels at the left lateral bladder neck and resect additional tumor regrowth.

The patient followed up in clinic 1 month later, and after an in-depth discussion of his clinical course, he decided to undergo surgical extirpation of the bladder and prostate with creation of an ileal conduit urinary diversion. When reviewing the preoperative MRI there was loss of fat plane between the prostate and rectum, therefore colorectal surgery was consulted to assist in rectal dissection. He also underwent preoperative colonoscopy in order to rule out invasion into the rectum and screen the remainder of the colon. The patient underwent open radical cystoprostatectomy with ileal conduit urinary diversion and intraoperative angiogram of the involved bowel segment and omental flap interposition. He had intraoperative proctoscopy by colorectal surgery to ensure no rectal injury was present. He was discharged on postoperative day 4 after progressing without incident. The pathology report from his extirpative surgery revealed a 1.8 cm leiomyoma involving the submucosal bladder wall surrounding the left ureteral orifice. Incidental finding of High Grade Prostatic Intraepithelial Neoplasia was noted on the prostate specimen. The findings did not extend through the bladder wall into the perivesical or periprostatic adipose. Margins were negative. The patient was evaluated 8 weeks postoperatively and has had an uncomplicated postoperative course to date. He has been satisfied by his improved functional status and is to be followed with serial renal ultrasound in 6 months to ensure no obstruction at the uretero-ileal anastomoses.

## 3. Discussion

**Figure 2:**
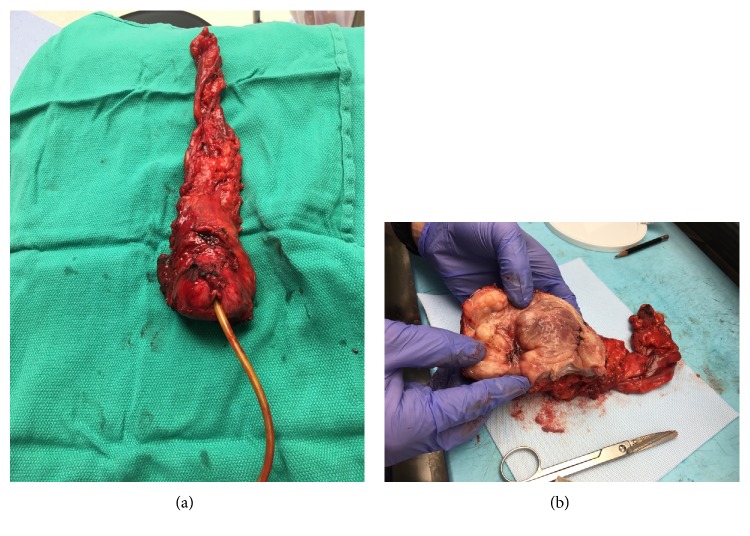
(a) Radical prostatectomy gross specimen. Prostate measured 4 cm from anterior to posterior. Prostate pathology showed high focal intraepithelial neoplasia, acute and chronic inflammation, and previous biopsy site changes. (b) Gross specimen of bivalved bladder. Submucosal leiomyoma measuring 1.8 cm is noted in the area of the left ureteral orifice. The leiomyoma was obstructing the left ureteral orifice. Bladder measured 5 × 5 × 2.5 cm.

Leiomyoma of the bladder is a very rare, benign tumor; however, it can be locally invasive and incur significant morbidity if left untreated. Leiomyomas are classified into 3 groups based on location: intravesical, intramural and extravesical [4, 5]. The most common presenting symptoms are obstructive voiding (49%), irritative symptoms (38%), hematuria (11%), and 20% were asymptomatic [6]. The patient in this case presented initially with hematuria prompting initial TURBT revealing leiomyoma of the bladder. The case differs from the previous literature because leiomyoma of the bladder is typically cured by visually complete excision and does not recur. There are around 250 cases of bladder leiomyoma reported [1], and recurrence has never been observed after either complete extirpation of the bladder or complete TUR. Silva-Ramos et al. performed an analysis of 90 cases of leiomyomas. On follow-up of the cases published there has been no recurrence up to 20 years after surgery and no malignant transformation [7]. Our patient's course is in contrast to the published literature because he underwent four visually complete resections endoscopically, but exhibited recurrence after each. After a long course of repeated endoscopic management the patient elected to undergo surgical extirpation of the bladder, prostate, and creation of an ileal conduit urinary diversion. Although only 1.8 cm of residual was noted, it is likely that the unique location of the patient's tumor made it more difficult to completely resect endoscopically which may explain his numerous recurrences (Figure 2(b)). This demonstrates that endoscopic resection of bladder leiomyomas is not always curative even if no gross tumor remains, and bladder leiomyomas can recur after numerous visually complete resections.
